# Machine learning and molecular dynamics simulations predict potential TGR5 agonists for type 2 diabetes treatment

**DOI:** 10.3389/fchem.2024.1503593

**Published:** 2025-01-09

**Authors:** Ojochenemi A. Enejoh, Chinelo H. Okonkwo, Hector Nortey, Olalekan A. Kemiki, Ainembabazi Moses, Florence N. Mbaoji, Abdulrazak S. Yusuf, Olaitan I. Awe

**Affiliations:** ^1^ Genetics, Genomics and Bioinformatics Department, National Biotechnology Research and Development Agency, Abuja, Nigeria; ^2^ Department of Pharmacy, National Hospital Abuja, Abuja, Nigeria; ^3^ Department of Clinical Pathology, Noguchi Memorial Institute for Medical Research, College of Health Science, University of Ghana, Accra, Ghana; ^4^ Molecular and Tissue Culture Laboratory, Babcock University, Ilisan-remo, Ogun State, Nigeria; ^5^ African Centers of Excellence in Bioinformatics and data intensive sciences, Department of Immunology and Microbiology, Makerere University, Makerere, Uganda; ^6^ Infectious Disease Institute (IDI), Makerere University, Kampala, Uganda; ^7^ Department of Pharmacology and Toxicology, Faculty of Pharmaceutical Sciences, University of Nigeria, Nsukka, Enugu, Nigeria; ^8^ Department of Biochemistry, Faculty of Basic Health Science, Bayero University, Kano, Nigeria; ^9^ African Society for Bioinformatics and Computational Biology, Cape Town, South Africa

**Keywords:** TGR5, type 2 diabetes, machine learning, molecular docking, molecular dynamics simulation, COCONUT database

## Abstract

**Introduction:**

Treatment of type 2 diabetes (T2D) remains a significant challenge because of its multifactorial nature and complex metabolic pathways. There is growing interest in finding new therapeutic targets that could lead to safer and more effective treatment options. Takeda G protein-coupled receptor 5 (TGR5) is a promising antidiabetic target that plays a key role in metabolic regulation, especially in glucose homeostasis and energy expenditure. TGR5 agonists are attractive candidates for T2D therapy because of their ability to improve glycemic control. This study used machine learning-based models (ML), molecular docking (MD), and molecular dynamics simulations (MDS) to explore novel small molecules as potential TGR5 agonists.

**Methods:**

Bioactivity data for known TGR5 agonists were obtained from the ChEMBL database. The dataset was cleaned and molecular descriptors based on Lipinski’s rule of five were selected as input features for the ML model, which was built using the Random Forest algorithm. The optimized ML model was used to screen the COCONUT database and predict potential TGR5 agonists based on their molecular features. 6,656 compounds predicted from the COCONUT database were docked within the active site of TGR5 to calculate their binding energies. The four top-scoring compounds with the lowest binding energies were selected and their activities were compared to those of the co-crystallized ligand. A 100 ns MDS was used to assess the binding stability of the compounds to TGR5.

**Results:**

Molecular docking results showed that the lead compounds had a stronger affinity for TGR5 than the cocrystallized ligand. MDS revealed that the lead compounds were stable within the TGR5 binding pocket.

**Discussion:**

The combination of ML, MD, and MDS provides a powerful approach for predicting new TGR5 agonists that can be optimised for T2D treatment.

## 1 Introduction

Type 2 diabetes (T2D) is an escalating metabolic disorder of global health concern (Ong et al., 2023). This disease is characterised by persistent hyperglycaemia due to insulin resistance and an eventual decline in pancreatic β-cell function (Bhatti et al., 2022; Deol and Bashir, 2024). In 2017, approximately 462 million people worldwide were affected by T2DM (Abdul Basith Khan et al., 2020). Individuals with T2DM are susceptible to long-term complications, including cardiovascular disease, neuropathy, retinopathy, and kidney failure, which lead to significant morbidity and mortality (DeFronzo et al., 2015; Chatterjee et al., 2017; Sharma et al., 2024). Although genetic factors have been correlated with the pathogenesis of the disease, environmental factors (consumption of unhealthy diet, reduced physical activity, and obesity) enhance pathophysiological anomalies associated with defective glucose homeostasis (Abolo et al., 2024; Mansour et al., 2023; Ikwuka et al., 2023).

Takeda G protein-coupled receptor 5 (TGR5) is a member of the G protein-coupled receptor (GPCR), class A (Thomas et al., 2008; Guo et al., 2016). TGR5 has emerged as a promising target in the context of T2DM owing to its involvement in glucose homeostasis, energy expenditure, and anti-inflammatory pathways (Sato et al., 2007; Bhimanwar and Mittal, 2021). TGR5 is activated by bile acids and plays a crucial role in regulating metabolic processes in various tissues, including the liver, pancreas, and adipose tissue (Lun et al., 2023)

Despite its potential, development of a TGR5 agonist as a therapeutic agent has faced several challenges. Identifying selective and potent TGR5 agonists is complicated by the structural flexibility of the receptor and the need for compounds that can cross biological membranes and exhibit favourable pharmacokinetic properties. Moreover, many identified TGR5 agonists have off-target effects or are associated with safety concerns, particularly regarding their impact on the gastrointestinal system.

Recent studies have shown the use of multi-omics and transcriptomic data integration approaches to predict potential biomarkers for diseases (Alaya et al., 2024; Ben Aribi et al., 2024; Chikwambi et al., 2023; El Abed et al., 2023; Nzungize et al., 2022; Wesonga and Awe, 2022), as well as to understand disease susceptibility (Nyamari et al., 2023). Other studies have also provided intriguing insights into viral evolution, diversity, and variation using computational approaches (Awe et al., 2023; Mwanga et al., 2023; Obura et al., 2022; Oluwagbemi and Awe, 2018).

In the field of drug discovery, machine learning (ML), molecular docking (MD), and molecular dynamics simulations (MDS) have revolutionized the identification and optimization of novel drug candidates (Di Stefano et al., 2022; Sadybekov and Katritch, 2023). ML models can rapidly analyze vast chemical libraries and predict the bioactivity of compounds with high accuracy, thereby significantly reducing the time and cost associated with traditional drug discovery methods (Di Stefano et al., 2022; Bhimanwar et al., 2023). Molecular docking studies provide insight into the interactions between small molecules and their target receptors, enabling the identification of key binding interactions that contribute to receptor activation or inhibition (Mursal et al., 2024). MDS further refines these predictions by accounting for the dynamic nature of protein-ligand interactions, providing a more realistic assessment of a compound’s stability and efficacy (Ogbodo et al., 2023; Brueckner et al., 2024).

Recent advancements in machine learning have led to the development of sophisticated algorithms capable of learning complex patterns in chemical data, enabling the prediction of bioactive compounds from diverse chemical spaces (van Heerden et al., 2023). In the context of TGR5 agonist discovery, several studies have applied ML techniques to screen compound libraries and predict potential agonists (Qin et al., 2023). Furthermore, molecular docking has been used to explore the binding interactions of the predicted agonists with TGR5 (Sindhu and Srinivasan, 2015). This study aims to contribute to the growing field of TGR5-targeted therapies by providing a systematic and validated approach for the discovery of potential TGR5 agonists.

## 2 Methods

### 2.1 Machine learning

**FIGURE 1:**
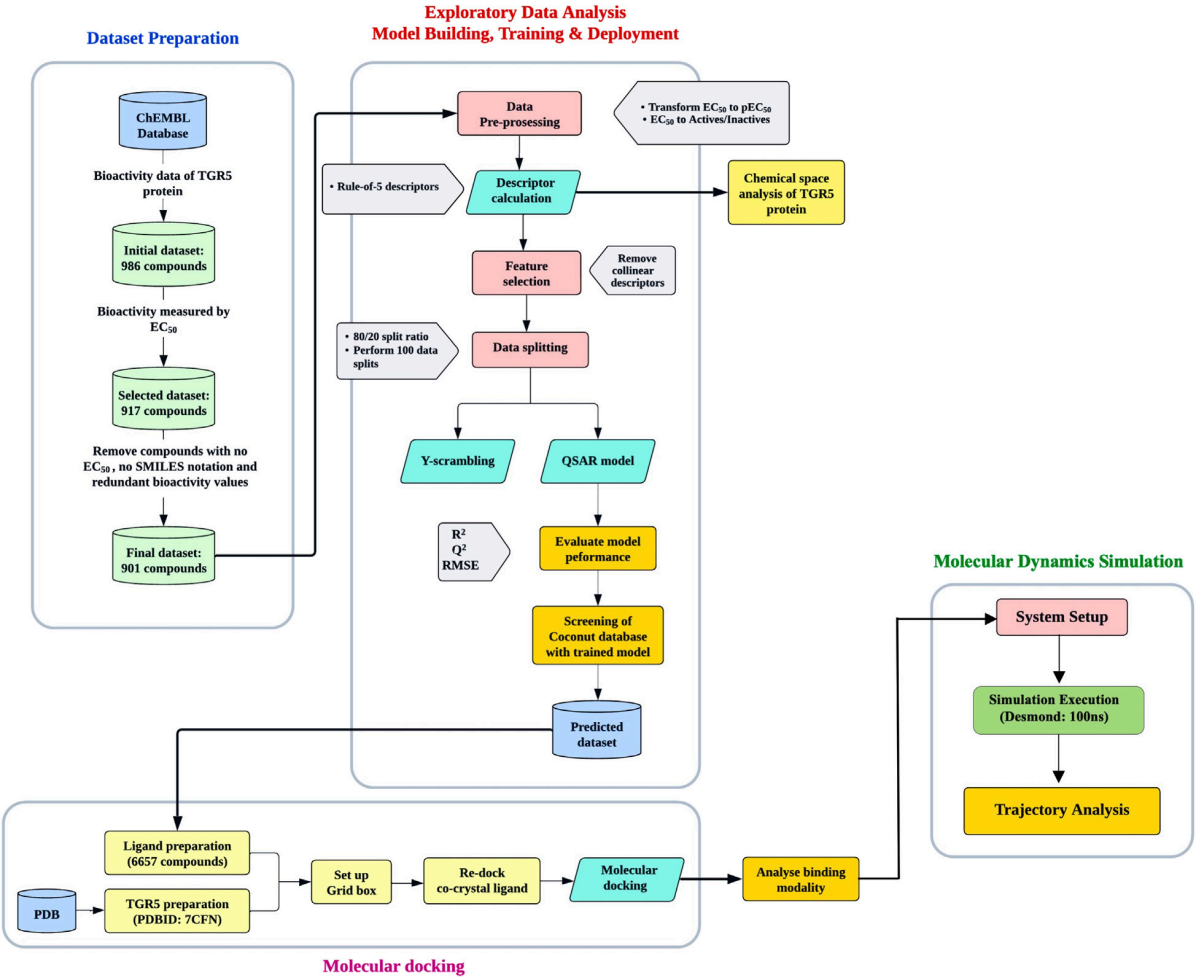
The workflow pipeline used in this study. It is divided into three parts: Machine learning, molecular docking, and molecular dynamics simulation.

The workflow pipeline used in this study is summarised in Figure 1.

#### 2.1.1 Data collection and preprocessing

Bioactivity data of compounds with biological activity for TGR5 (CHEMBL5409), which are expressed as EC_50_ values in nM (nanomolar), was downloaded from the ChEMBL database (https://www.ebi.ac.uk/chembl/) (Bento et al., 2014; Gaulton et al., 2017). ChEMBL is a comprehensive, curated bioactivity database containing information on molecule-target interactions extracted from published literature. The data were cleaned, which involved removing any compounds with missing EC_50_ values, those without smile notation and redundant bioactivity values. For the data preprocessing step, compounds were classified as active if their values were less than 1,000 nM, and inactive if they were more than 10,000 nM. Values between 1,000 and 10,000 nM were considered intermediate. The intermediate category was removed, leading to an exploratory data analysis that focused only on active and inactive compounds.

#### 2.1.2 Exploratory data analysis

The dataset includes chemical names and corresponding SMILES notations, which provide information about the molecular structure used to calculate the molecular descriptors. The drug-likeness of the compounds was assessed based on the pharmacokinetic parameters of absorption, distribution, metabolism, and excretion (ADME). Lipinski’s Rule of Five (Ro5), which states that a compound should have a molecular weight of less than 500 Da, an octanol-water partition coefficient (LogP) of less than 5, fewer than 5 hydrogen bond donors, and fewer than 10 hydrogen bond acceptors, was used to compute the molecular descriptors (Lipinski et al., 2012). Ro5 provides insight into a compound’s potential for absorption in the body, distribution to the appropriate target tissue or organ, metabolism, and eventual excretion from the body. To ensure a more uniform distribution of EC_50_ data, EC_50_ values were converted to a negative logarithmic scale (i.e., -log10), resulting in the pEC_50_ metric.

#### 2.1.3 Model building, training, and deployment

Selected molecular descriptors were used as input features to build the model. The model was built using the Random Forest algorithm to distinguish between agonists and nonagonists. Fingerprint descriptors were generated using PaDEL (Yap, 2011), and data matrices were prepared accordingly. Features with low variance were removed from the dataset and divided in an 80:20 ratio for training purposes. To prevent potential bias arising from a single data split in constructing predictive models, the models were developed using 100 independent data splits (Puzyn et al., 2011). The optimised ML model was deployed in the form of an offline application using Streamlit to screen the COCONUT (COlleCtion of Open Natural prodUcTs) database (https://coconut.naturalproducts.net), predicting potential TGR5 agonists based on their molecular features (Sorokina et al., 2021). More than four hundred thousand natural compounds that have been sourced from open and free sources are stored in the COCONUT database (Capecchi and Reymond, 2021).

### 2.2 Molecular docking

#### 2.2.1 Ligand and receptor preparation

##### 2.2.1.1 Ligand Preparation

The SMILES of these compounds were obtained and converted to the 2D format using Datawarrior and prepared using the LigPrep module in Schrödinger. This tool was employed to generate the most probable protonation states at physiological pH (7.0 ± 2.0), ensure the correct stereochemistry, and minimise the energy of the ligand structures using the OPLS4 force field.

##### 2.2.1.2 Protein preparation

The crystal structure of the TGR5 receptor (PDBID:7CFN) (Yang et al., 2020) was downloaded from the Protein Data Bank (PDB) and imported into Maestro (Schrodinger, 2021). The Protein Preparation Wizard was used to prepare the downloaded protein, which involved removing water molecules beyond 5 Å from the binding site, adding missing hydrogen atoms, assigning proper bond orders, adjusting protonation states of ionisable residues, and minimising the receptor using the OPLS4 force field to relieve steric clashes and optimise geometry. A grid box was generated around the active site where the ligands were docked.

#### 2.2.2 Molecular docking

The prepared ligands were docked into the active site of TGR5 using the Glide tool in Schrödinger (Schrodinger, 2021). SP (standard precision) and XP (extra-precision) protocols were applied. The results were analysed by examining their binding energies to TGR5. 295 compounds had lower binding energies compared to the co-crystallised ligand. The top 4 ligands with the lowest docking scores were selected for succeeding molecular dynamics simulations.

#### 2.2.3 Validation of docking protocol

The docking protocol was validated by re-docking the co-crystallised ligand into the active site of the TGR5 protein and calculating the RMSD of the two poses (Shivanika et al., 2020).

### 2.3 Molecular dynamics simulation

#### 2.3.1 System setup

The simulation system was prepared using the Desmond System Setup tool. The TGR5-ligand complexes obtained from docking studies were embedded in a POPC (300k) membrane bilayer. Appropriate ions were added to neutralise the system. Energy minimisation was performed to remove any steric clashes, followed by equilibration to stabilise the temperature and pressure of the system.

The protein-ligand complex was solvated in an orthorhombic simulation box filled with explicit TIP3P water molecules. The buffer distance between the complex and edge of the simulation box was set to 10 Å to avoid boundary effects, and 0.15 M NaCl was added to neutralise the system and mimic physiological conditions. The OPLS4 force field was applied to describe the interactions between atoms in the system, including the protein, ligand, and solvent molecules. Before carrying out MD simulation, energy minimisation was performed to remove any steric clashes or bad contacts introduced during the system setup.

#### 2.3.2 Simulation run

The simulations were conducted under a constant number of particles, pressure (1 atm), and temperature (300 K) using the Desmond module of the Schrodinger software. The model system was relaxed before simulation and equilibrated, after which a 100 ns production run was carried out, with coordinates recorded every 100 ps for subsequent analysis. The simulation trajectory was monitored to ensure system stability throughout the run. MDS was carried out on a GPU-enabled Linux operating system.

#### 2.3.3 Post-simulation trajectory analysis

The trajectory was analysed to assess the binding stability, interaction energy, and conformational dynamics of the TGR5-ligand complexes. This analysis helps to identify the most promising TGR5 agonist for further experimental validation. The simulation trajectories were analysed using the simulation interaction diagram tool in Schrödinger. Key metrics included:

- Root Mean Square Deviation (RMSD): To evaluate the stability of the protein-ligand complex.
- Root Mean Square Fluctuation (RMSF): To analyse the flexibility of individual residues in the receptor.
- Radius of gyration (RoG): To measure the extendedness of a ligand, it is equivalent to its principal moment of inertia.
- Intramolecular hydrogen bonds (intraHB): the number of internal hydrogen bonds within a ligand molecule.
- Ligand-Protein interactions: To monitor the types of interactions (e.g., hydrogen bonds, hydrophobic contacts) between the ligand and receptor throughout the simulation.

## 3 Results

### 3.1 Chemical space analysis of TGR5 activators

**FIGURE 2:**
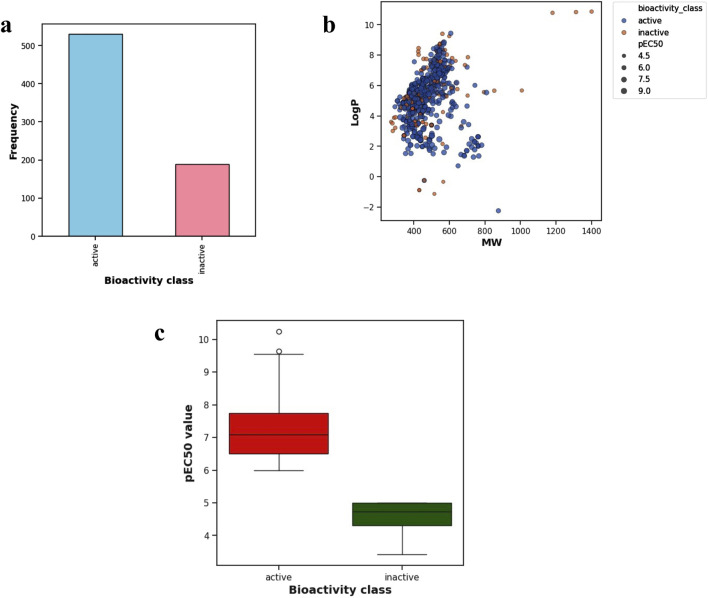
**(A)** Frequency plot of the two bioactivity classes **(B)** scatter plot of MW versus LogP and **(C)** box plot showing the distribution of pEC_50_ values of the two bioactivity classes.

A total of 518 active, 187 inactive, and 190 intermediate compounds were identified after the data preparation step (Figure 2A). The two bioactivity classes span similar chemical spaces, as shown by the scatter plot of MW vs. LogP (Figure 2B). Considering the pEC_50_ values (Figure 2C), the actives and inactives displayed statistically significant differences, which was to be expected since threshold values (EC_50_ < 1000 nM = Actives and EC_50_ > 10,000 nM = Inactives, corresponding to pEC_50_ > 6 = Actives and pEC_50_ < 5 = Inactives) were used to define actives and inactives.

### 3.2 Lipinski’s descriptors

**FIGURE 3:**
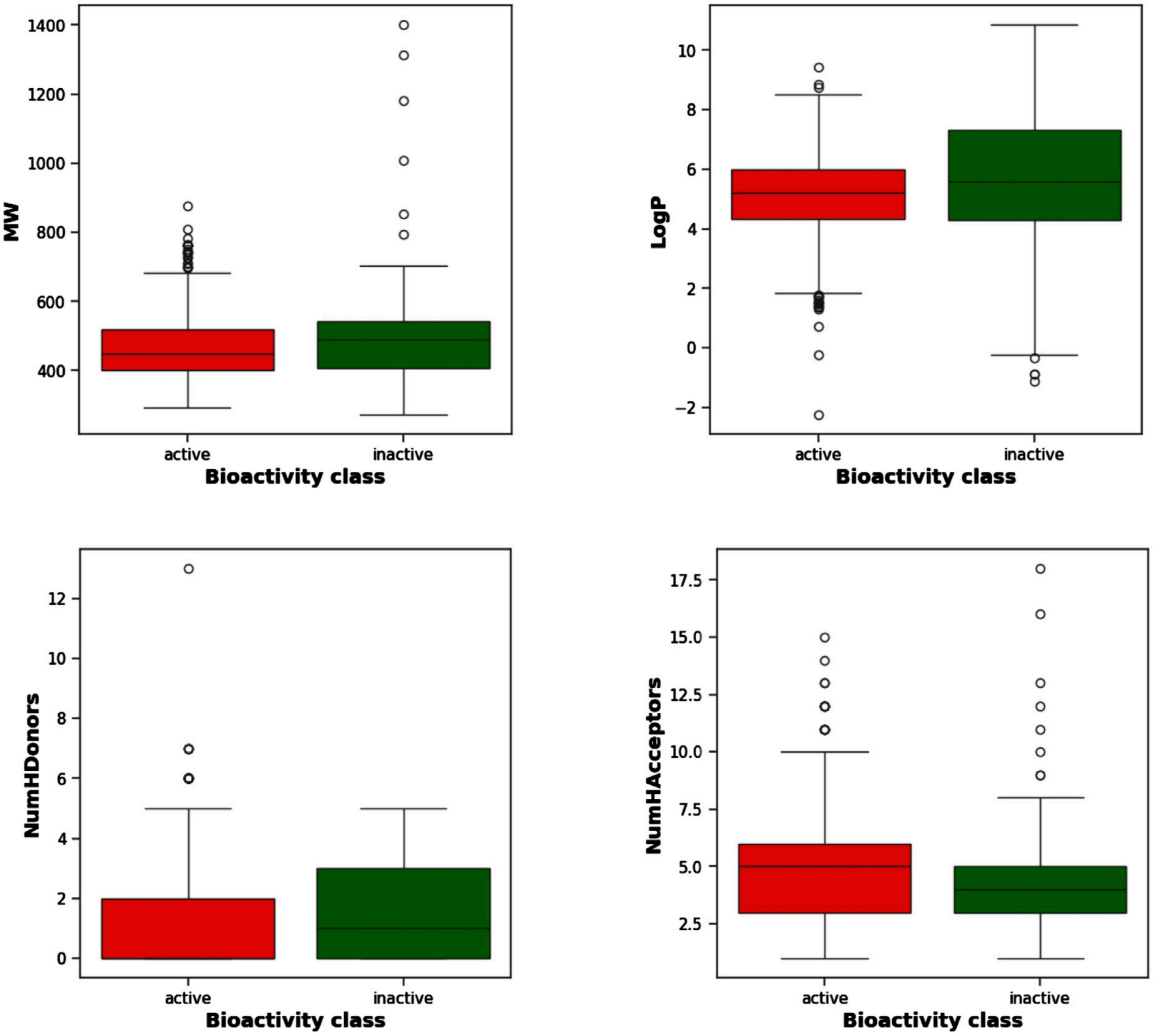
Box plots of TGR5 agonists using Lipinski’s descriptors: LogP, molecular weight (MW), number of hydrogen acceptors (NumHAcceptors), and number of hydrogen donors (NumHDonors).

Figure 3 displays the box plots of Lipinski’s descriptors. Of the four Lipinski descriptors (MW, LogP, NumHDonors, and NumHAcceptors), only LogP exhibited no difference between the actives and inactives, while the other three descriptors (MW, NumHDonors, and NumHAcceptors) showed statistically significant differences between the active and inactive groups (Table 1).

**TABLE 1:** Statistical analysis | Mann-Whitney U test.

	Descriptor	Statistics	P value	Alpha	Interpretation
1	pEC_50_	100,170.0	7.281833e-93	0.05	Different distribution (reject H0)
2	Molecular weight	44,041.0	0.013695	0.05	Different distribution (reject H0)
3	LogP	40,977.0	0.000203	0.05	Different distribution (reject H0)
4	NumHDonors	40,126.5	0.000017	0.05	Different distribution (reject H0)
5	NumHAcceptors	56,090.5	0.012607	0.05	Different distribution (reject H0)

### 3.3 Machine learning model to predict TGR5 agonists

**FIGURE 4:**
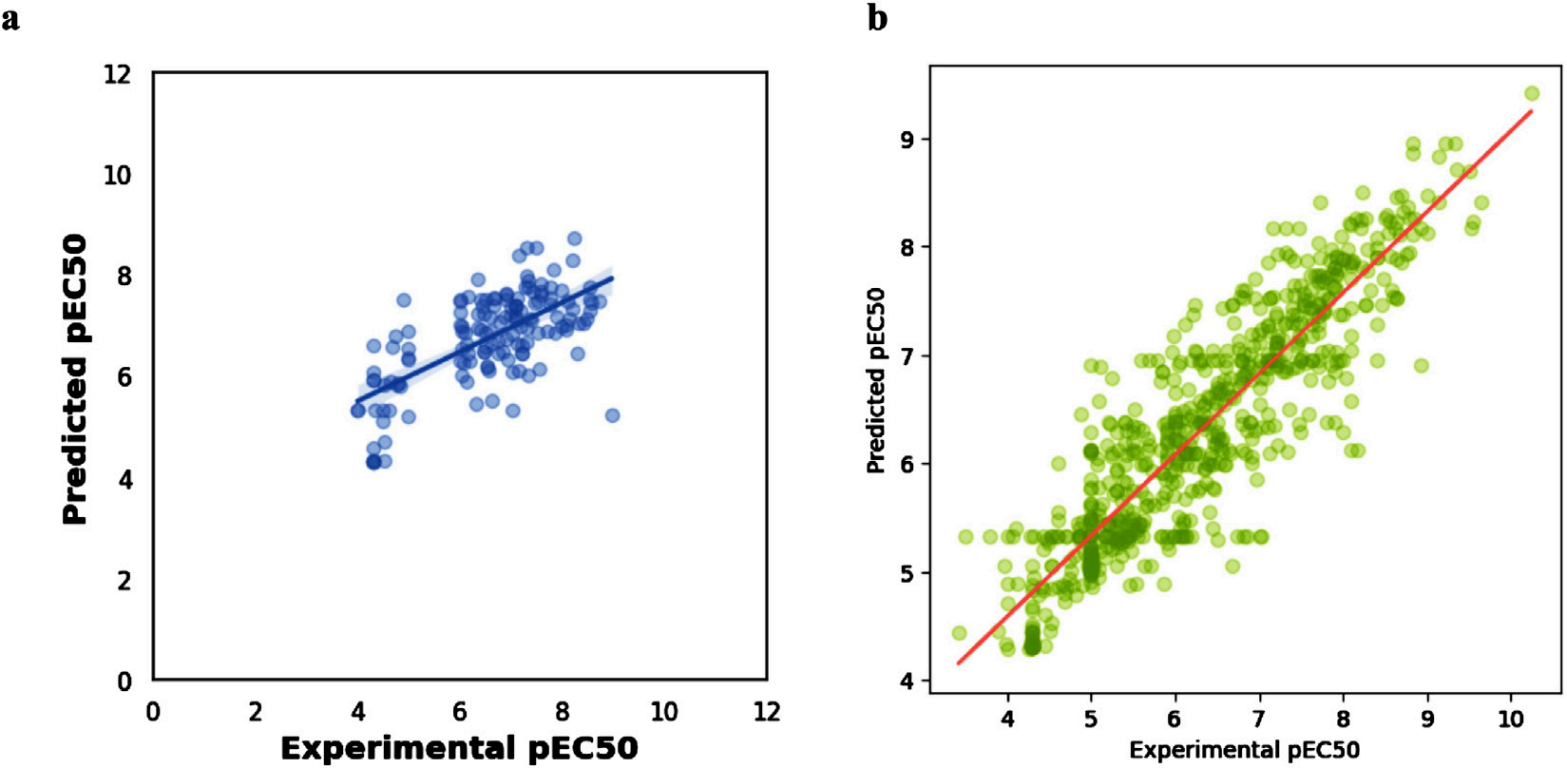
Scatter plots of **(A)** regression model using random forest algorithm **(B)**: experimental vs. predicted pEC_50_ for training data.

**FIGURE 5:**
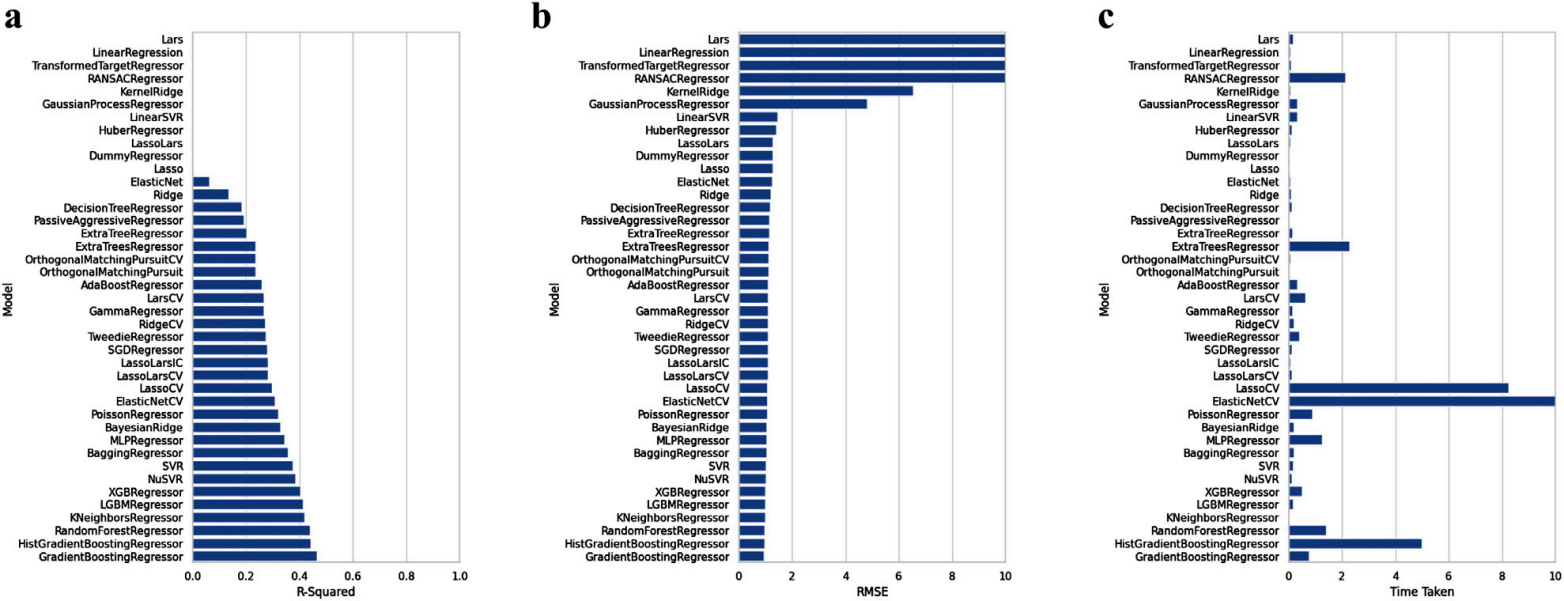
Comparison of the performance of machine learning algorithms against R-squared **(A)**, RMSE **(B)**, and time taken **(C)**, respectively.

Figure 4A shows the resulting scatterplot of the regression model built using the random forest algorithm. The regression model score (r^2^) is given as 0.40. Figure 4B shows the predicted pEC_50_ values of the training data. The mean squared error (MSE) and coefficient of determination (R^2^) for model performance are 0.34 and 0.80, respectively. Figure 5 shows a visual representation of the model performance. This shows that the model had a high r^2^ and low root-mean-square error value.

ML-based prediction identified 340,364 compounds with potential activity towards TGR5 having EC_50_ values ranging from 4.0–6.9. Only compounds with EC_50_ values between 4.0 and 4.9 were selected for docking, yielding 6,656 compounds in total. The bioactivity predictions of just the four top-scoring compounds selected in this study are displayed in Table 2.

**TABLE 2:** Predicted EC_50_ values of the four top-scoring compounds from the screening of the COCONUT database.

COCONUT ID	Predicted pEC_50_
CNP0209363	4.99
CNP0424850	4.97
CNP0417335	4.91
CNP0224616	4.90

### 3.4 Molecular docking reveals the binding energy of lead compounds

**FIGURE 6:**
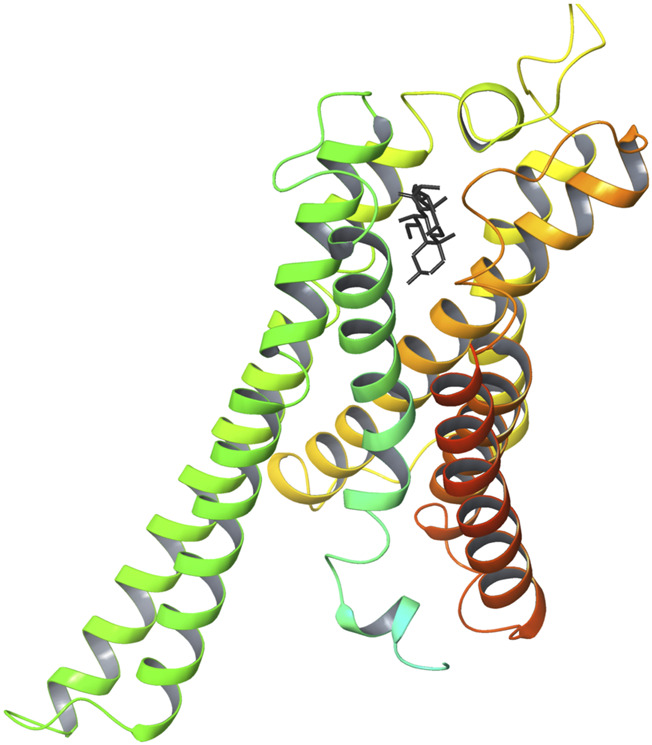
3D structure of TGR5 protein (7CFN) in complex with its co-crystallized ligand (INT-777).

**FIGURE 7:**
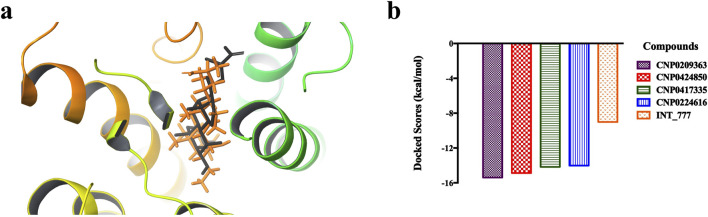
**(A)** Validation of docking protocol. The co-crystalized ligand (in grey) was redocked (in orange) into the active site of the TGR5 protein and superimposed. The calculated RMSD value between the native and re-docked pose was calulated as 1.42 Å. **(B)** Bar chart showing the docked scores of the lead compounds and the co-crystallized ligand.

**FIGURE 8:**
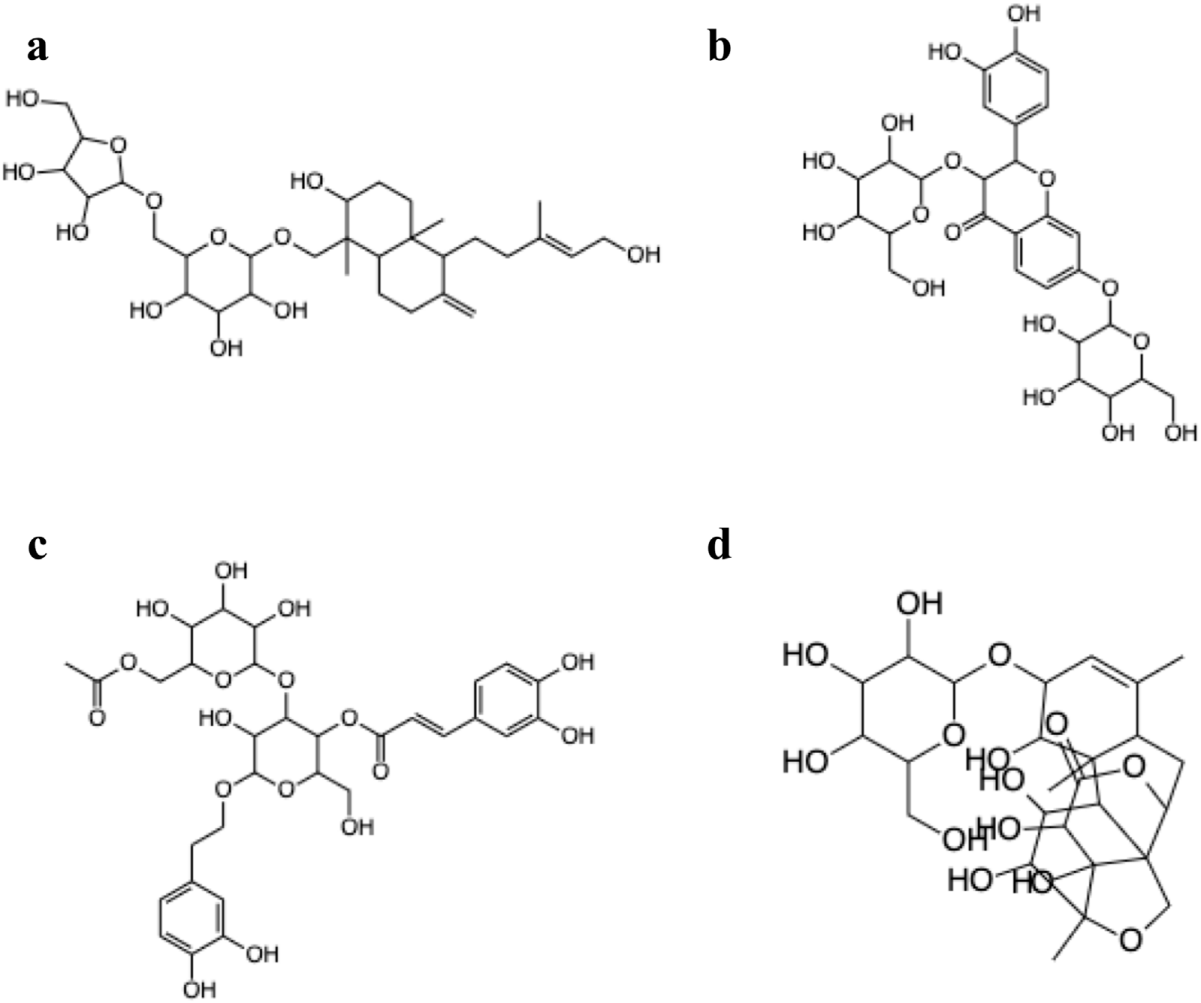
2D structures of the lead compounds from the COCONUT database **(A)** CNP0209363, **(B)** CNP0424850, **(C)** CNP0417335, **(D)** CNP0224616.

Figure 6 shows the 3D structure of TGR5 protein downloaded from the protein data bank (PDBID: 7CFN) in complex with its co-crystallized ligand. After re-docking the co-crystalized ligand into the TGR5 active site, the calculated RMSD value between the docked and re-docked pose was given as 1.42 Å (Figure 7A). Figure 7B shows the docked scores of the top four-scoring compounds (also referred to as lead compounds), represented by their COCONUT IDs, CNP0209363, CNP0424850, CNP0417335, CNP0224616, and co-crystalized ligand, given as −15.39, −14.87, −14.17, −14.01, and −9.01 kcal/mol, respectively. Figure 8 shows the 2D structure of the lead compounds. All the lead compounds contain an acetal/aminal-like group (X-CH(R)-Y, where X, Y are N, S, or O) that may be acid/base labile, releasing an aldehyde. CNP0417335 and CNP0224616 have an ester group and may undergo hydrolysis at high or low pH.

**FIGURE 9:**
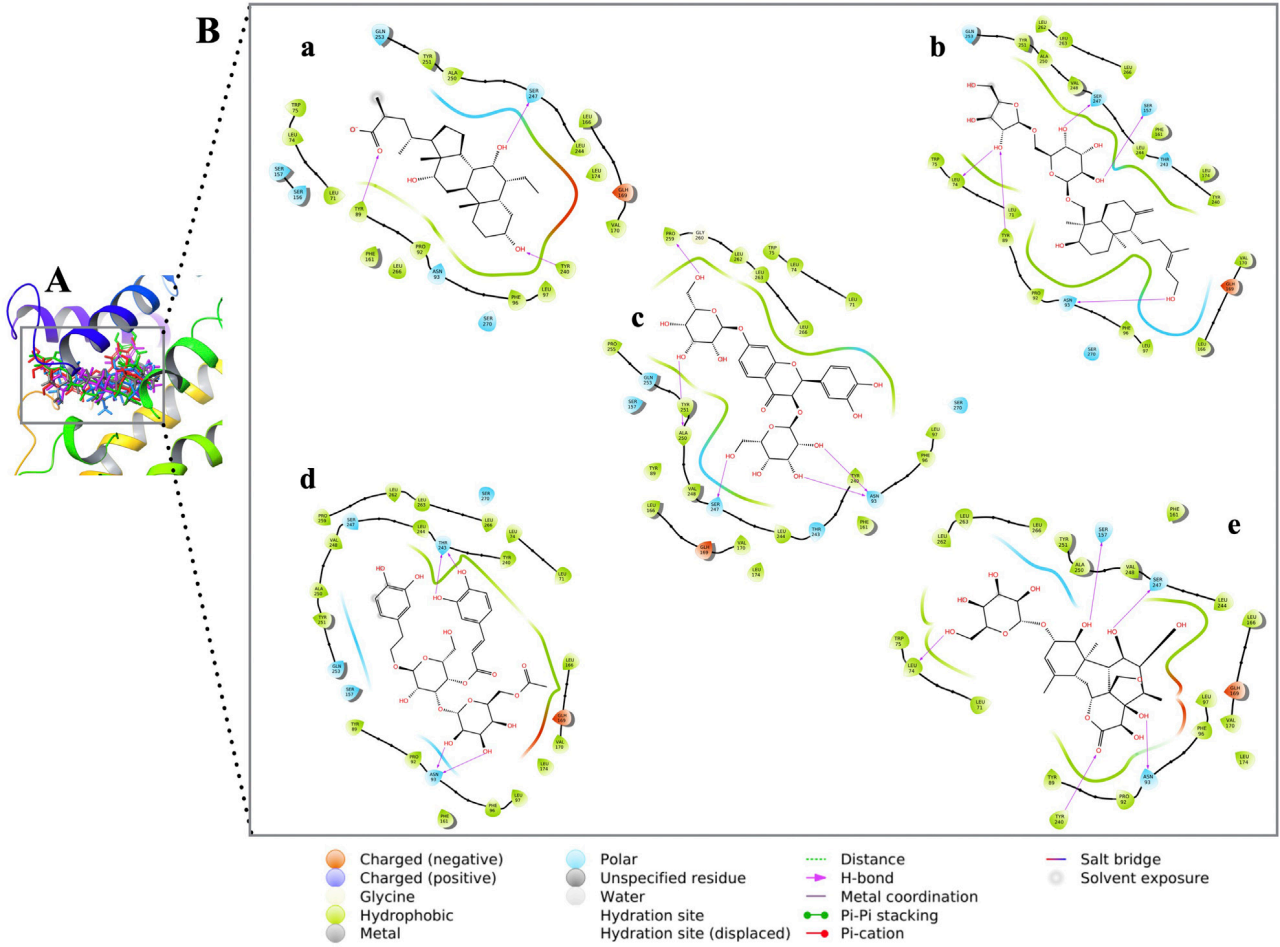
**(A)** 3D conformations of the compounds within the active site of TGR5, represented as coloured sticks: orange (INT-777), purple (CNP0209363), red (CNP0424850), green (CNP0417335) and blue (CNP0224616). **(B)** 2D molecular interaction diagrams of INT-777 (a), lead compounds (b. CNP0209363, c. CNP0424850, d. CNP0417335, e. CNP0224616) and TGR5. Hydrogen and hydrophobic bonds are shown. All the lead compounds show binding to residue Asn93.

Figure 9 shows the molecular interactions of the compounds with the amino acid residues found within the TGR5 binding pocket. An overview of the interactions is provided in Table 3. We observed that all the lead compounds formed hydrogen bonding with residue Asn93 during molecular docking.

**TABLE 3:** Overview of interacting amino acid residues and bond types of the compounds and TGR5 from molecular docking studies.

Compound name	Docking scores (kcal/mol)	Interacting amino acid residue	Bond type
INT-777	−9.01	Tyr 89 Tyr 240 Ser 247 Leu 71 Tyr 89 Pro 92 Phe 96 Leu 97 Tyr 240	Hydrogen bond Hydrogen bond Hydrogen bond Hydrophobic bond Hydrophobic bond Hydrophobic bond Hydrophobic bond Hydrophobic bond Hydrophobic bond
CNP0209363	−15.39	Ser 157 Ser 247 Asn 93 Leu 74 Tyr 89 Leu 71 Pro 92 Phe 96 Leu 97 Leu 166 Val 170 Tyr 240 Leu 244 Tyr 251	Hydrogen bond Hydrogen bond Hydrogen bond Hydrogen bond Hydrogen bond Hydrophobic bond Hydrophobic bond Hydrophobic bond Hydrophobic bond Hydrophobic bond Hydrophobic bond Hydrophobic bond Hydrophobic bond Hydrophobic bond
CNP0424850	−14.87	Ala 250 Ser 247 Asn 93 Pro 259 Pro 255 Val 248 Leu 244 Tyr 240 Leu 266 Leu 262 Leu 263 Leu 71 Leu 74	Hydrogen bond Hydrogen bond Hydrogen bond Hydrogen bond Hydrophobic bond Hydrophobic bond Hydrophobic bond Hydrophobic bond Hydrophobic bond Hydrophobic bond Hydrophobic bond Hydrophobic bond Hydrophobic bond
CNP0417335	−14.14	Asn 93 Thr 243 Pro 259 Val 248 Ala 250 Tyr 251 Tyr 89 Pro 92 Phe 96 Leu 97 Leu 166 Tyr 240 Leu 262 Leu 71 Leu 74 Leu 266	Hydrogen bond Hydrogen bond Hydrophobic bond Hydrophobic bond Hydrophobic bond Hydrophobic bond Hydrophobic bond Hydrophobic bond Hydrophobic bond Hydrophobic bond Hydrophobic bond Hydrophobic bond Hydrophobic bond Hydrophobic bond Hydrophobic bond Hydrophobic bond
CNP0224616	−14.01	Ser 247 Leu 74 Asn 93 Tyr 240 Ser 157 Leu 71 Leu 262 Leu 263 Leu 266 Ala 250 Leu 244 Leu 97 Phe 96 Val 170 Tyr 89 Tyr 240 Pro 92 Leu 166	Hydrogen bond Hydrogen bond Hydrogen bond Hydrogen bond Hydrogen bond Hydrophobic bond Hydrophobic bond Hydrophobic bond Hydrophobic bond Hydrophobic bond Hydrophobic bond Hydrophobic bond Hydrophobic bond Hydrophobic bond Hydrophobic bond Hydrophobic bond Hydrophobic bond Hydrophobic bond

### 3.5 Molecular dynamics simulation

The kinetics of the TGR5-compound complex was investigated using molecular dynamics simulations to assess the bond configuration stability after the binding of lead compounds within the protein cavity. Simulations were conducted over a 100 ns period for the co-ligand (INT-777) and the four lead compounds. The thermodynamic stability of these complex systems was analysed using three key parameters: root mean square deviation (RMSD), root mean square fluctuation (RMSF), and radius of gyration (RoG), all of which were monitored throughout the molecular dynamics simulation.

**FIGURE 10:**
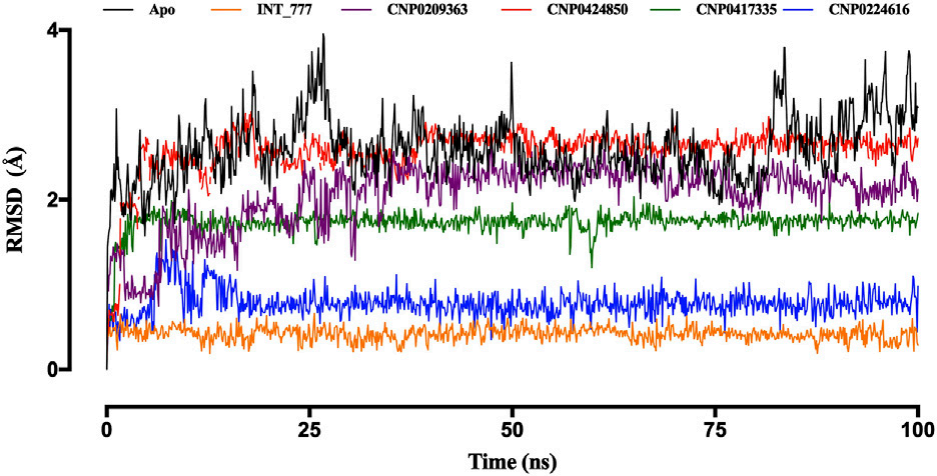
RMSD trajectories of TGR5 in the apo state and complex with INT-777, CNP0209363, CN 0424850, CNP0417335, and CNP0224616. Simulations were performed over a 100 ns (nanosecond) duration.

As shown in Figure 10, the RMSD of the TGR5 protein in its apo state demonstrated instability throughout the 100 ns simulation but became more stable upon binding to the co-ligand. Moreover, when the TGR5 protein was bound to the lead compounds, stability was observed within the range of 1 Å to 3 Å after the 25 ns mark.

Among the lead compounds, CNP0224616 exhibited the highest stability, with an RMSD value of approximately 0.8 Å, compared to the co-ligand (INT-777), which showed an RMSD of 0.6 Å CNP0209363, however, displayed lower stability, with its RMSD fluctuating between 1 Å and 2.2 Å, throughout the 100 ns simulation. Meanwhile, CNP0417335 and CNP0424850 stabilised after 10 ns and 25 ns, with respective RMSD values of about 1.8 Å and 2.7 Å (Figure 10).

**FIGURE 11:**
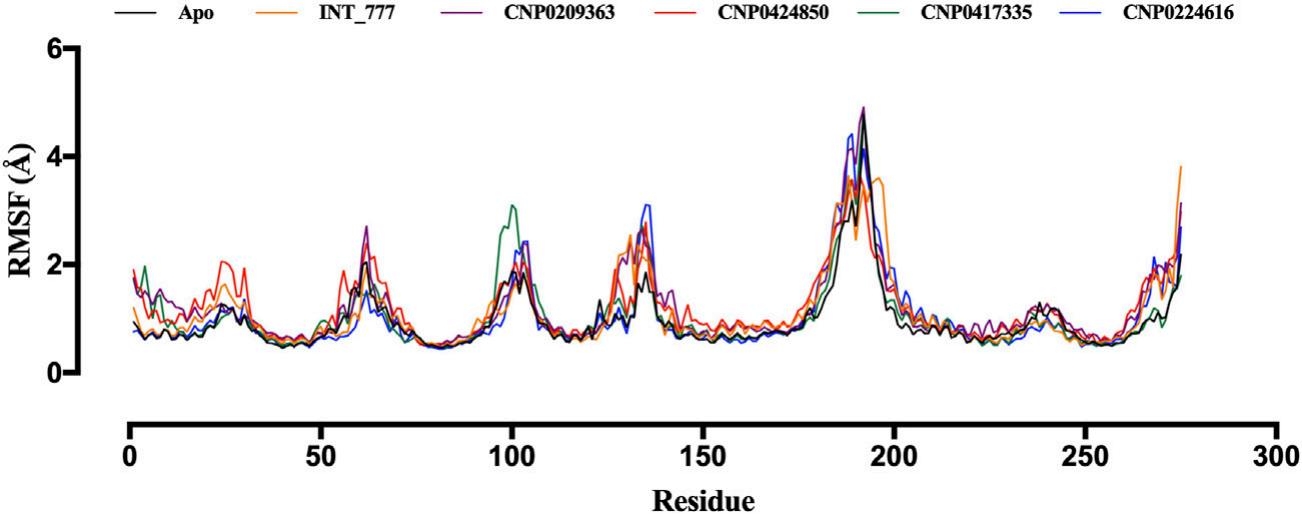
RMSF trajectories of TGR5 in the apo state and complex with INT-777, CNP0209363, CNP0424850, CNP0417335 and CNP0224616.

RMSF values provide insight into the magnitude of fluctuations for each residue in a protein; higher RMSF values indicate greater flexibility, and lower values suggest rigidity. Figure 11 shows that RMSF values between 2 Å and 5 Å were recorded during the 100 ns simulation, and the RMSF profiles of the co-ligand (INT-777) were comparable to those of the lead compounds.

**FIGURE 12:**
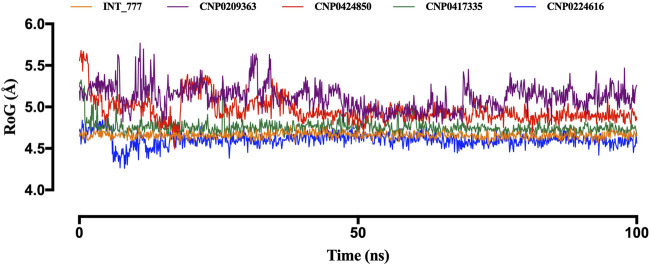
Radius of gyration (RoG) of the compounds in complex with TGR5.

Another parameter used to assess structural stability is the radius of gyration (RoG). Figure 12 illustrates the stability trends of the lead compounds and the co-ligand. CNP0417335 and CNP0224616 initially displayed slight fluctuations during the first 10 ns but stabilised for the remainder of the simulation, similar to the co-ligand, which remained stable around 4.6 Å. CNP0417335 and CNP0224616 stabilised at 4.7 Å and 4.6 Å, respectively. In contrast, CNP0424850 reached stability only after 50 ns, with a value close to 5 Å, while CNP0209363 showed little to no stability throughout the simulation.

**FIGURE 13:**
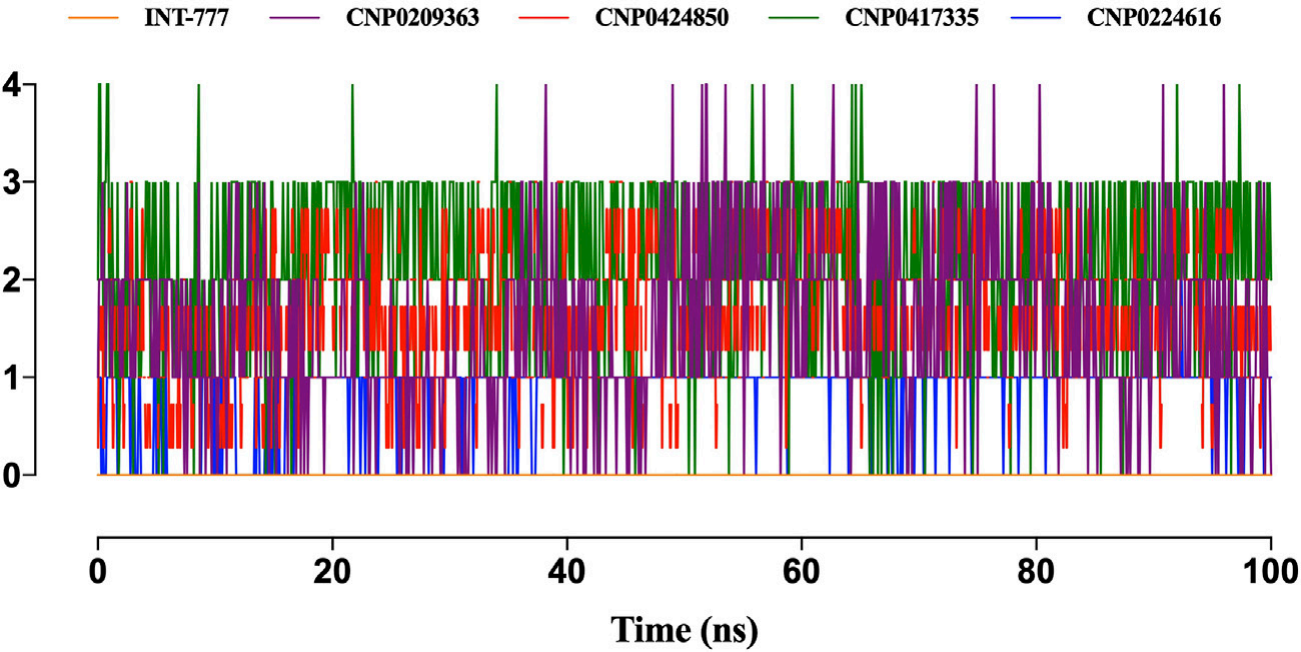
Only the lead compounds showed the presence of intramolecular hydrogen bonds present within the compounds. Only the lead compounds showed the presence of hydrogen bonds.

Figure 13 shows the intramolecular hydrogen bonds within the compounds. Only the lead compounds showed intramolecular hydrogen bonding up to a magnitude of 4. INT-777 showed no intramolecular hydrogen bonding during the simulation run.

**FIGURE 14:**
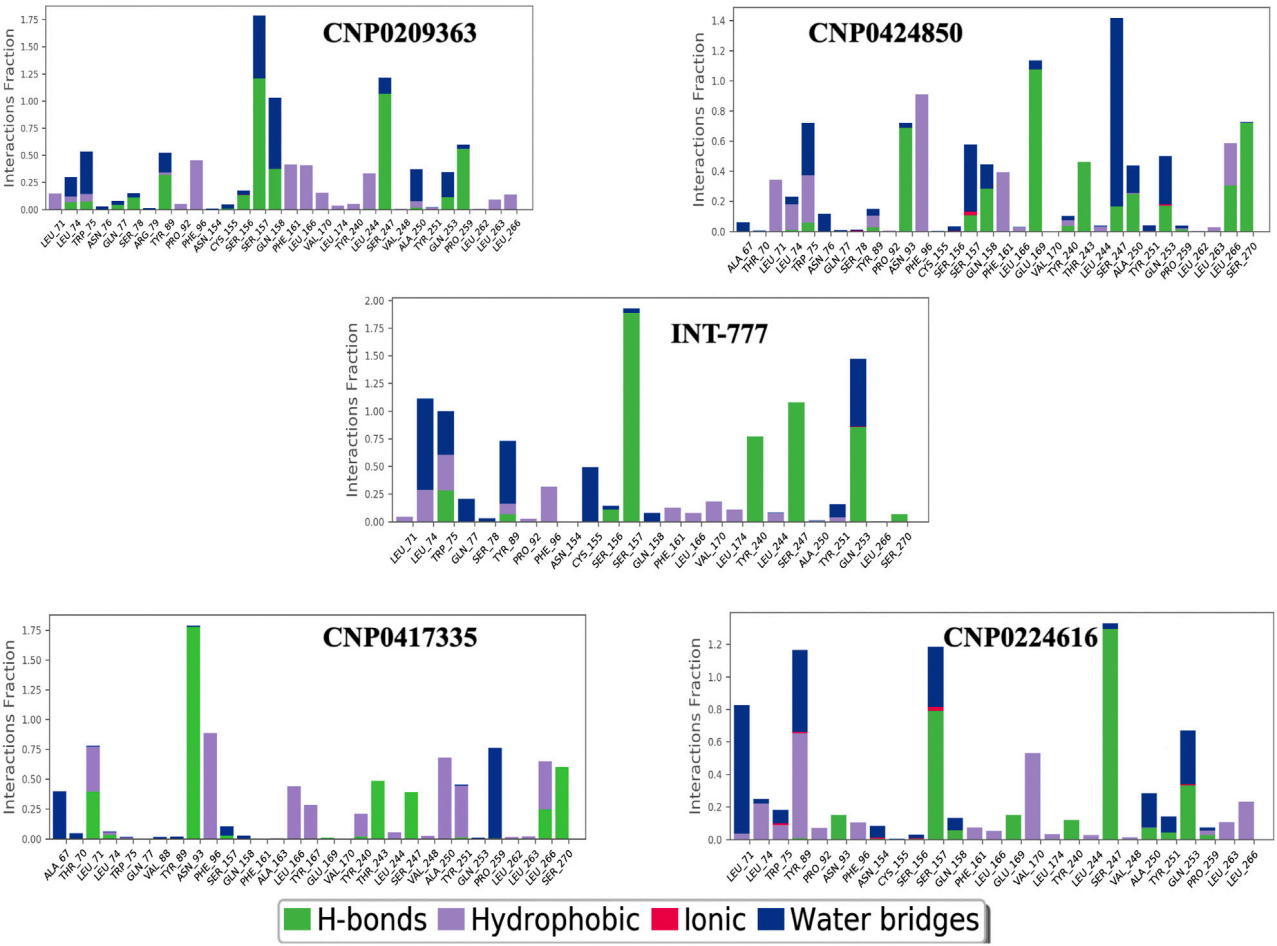
Protein-ligand contacts between TGR5 and the compounds during simulation run. The figure shows protein interactions with the ligand. The interactions are categorized into hydrogen bonds, hydrophobic bond, ionic bonds and water bridges. The bar charts show the amount of time a specific interaction is sustained.

Figure 14 shows the distribution of hydrogen bonds, hydrophobic bonds, ionic bonds and water bridges formed between the compounds and TGR5 during MDS. All the compounds showed binding to TGR5 via hydrogen bonds, hydrophobic bonds, and water bridges. Only INT-777, CNP0424850 and CNP0224616 showed ionic bonding via Gln253 residue.

**FIGURE 15:**
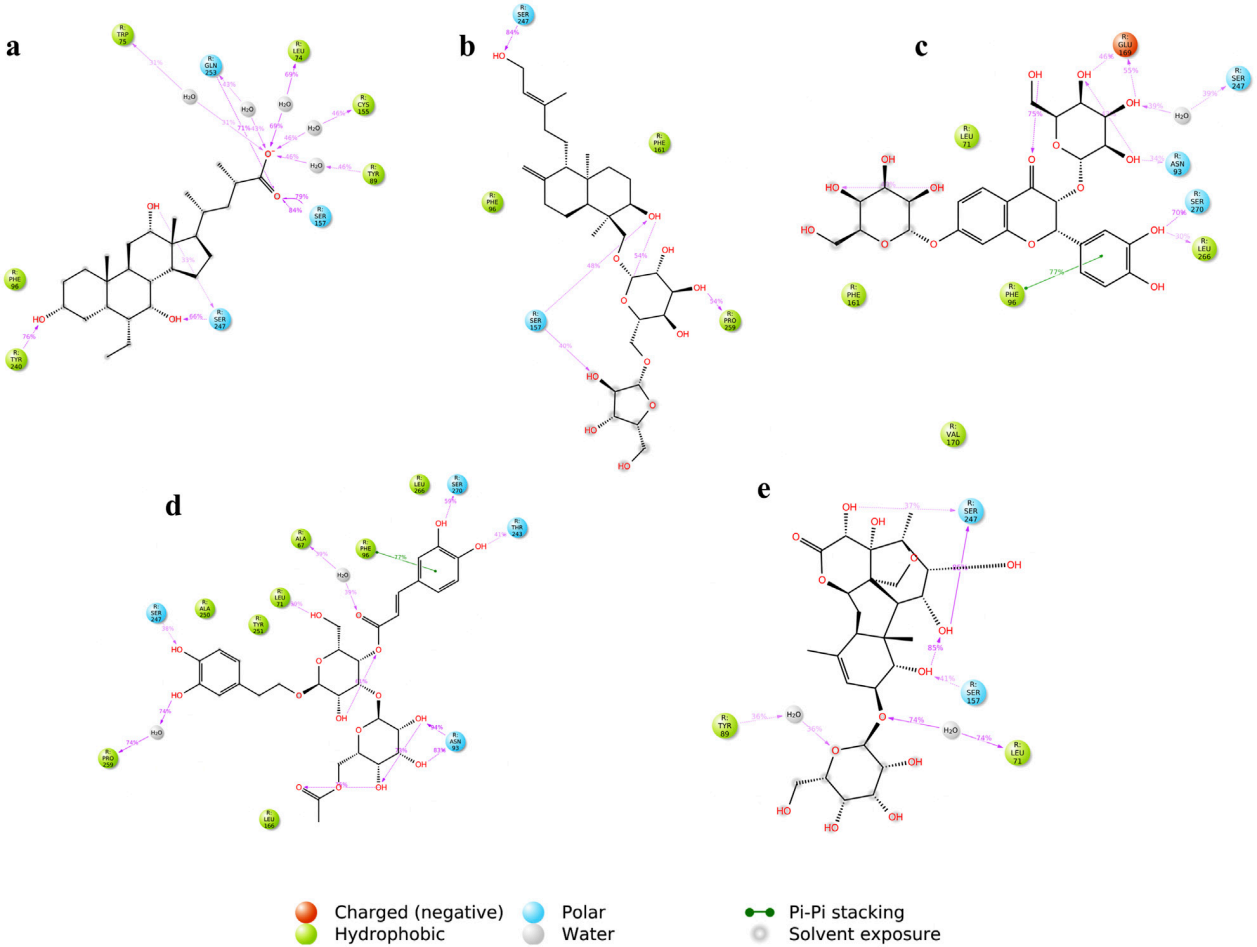
Ligand-protein contacts between the compounds and TGR5 during simulation run **(A)** INT-777; **(B)** CNP0209363; **(C)** CNP0424850; **(D)** CNP0417335; **(E)** CNP0224616. The figure shows a schematic detail of the interactions that occur for more than 30% of the simulation time.

Figure 15 shows the ligand-protein contacts made during simulation. An overview of the interactions is provided in Table 4. All the compounds showed binding with the Ser247 residue; this interaction was also observed from molecular docking studies. Only CNP0424850 and CNP0417335 showed pi-pi stacking.

**TABLE 4:** Overview of the interacting amino acid residues and bond types of the compounds and TGR5 during MDS.

Compound name	Interacting amino acid residue	Bond type
INT-777	Trp 75 Gln 253 Leu 74 Cys 155 Tyr 89 Ser 157 Ser 247 Tyr 240	Hydrogen bond Hydrogen bond Hydrogen bond Hydrogen bond Hydrogen bond Hydrogen bond Hydrogen bond Hydrogen bond
CNP0209363	Ser 157 Ser 247 Pro 259	Hydrogen bond Hydrogen bond Hydrogen bond
CNP0424850	Glu 169 Ser 247 Asn 93 Ser 270 Leu 266 Phe 96	Hydrogen bond Hydrogen bond Hydrogen bond Hydrogen bond Hydrogen bond π-π stacking
CNP0417335	Ser 270 Thr 243 Asn 93 Pro 259 Ser 247 Leu 71 Ala 67 Phe 96	Hydrogen bond Hydrogen bond Hydrogen bond Hydrogen bond Hydrogen bond Hydrogen bond Hydrogen bond π-π stacking
CNP0224616	Ser 247 Leu 71 Ser 157 Tyr 89	Hydrogen bond Hydrogen bond Hydrogen bond Hydrogen bond

## 4 Discussion

Type 2 diabetes is a leading cause of mortality (Abdul Basith Khan et al., 2020). Despite the therapeutic advancement in this disease management, imbalance in glucose homeostasis and energy expenditure associated with the progression of the diseases remains a challenge (Mirzadeh et al., 2022; Reed et al., 2021; Büsing et al., 2019; Stein et al., 2013). In this study, predictive machine learning-based models, molecular docking, and molecular dynamics simulation were used in the identification of TGR5 agonists for the management of type 2 diabetes.

Compounds with their corresponding EC_50_ values exhibiting biological activity towards TGR5 were downloaded from the ChEMBL database. Considering the biological activity of the compounds, they were characterised as either active or inactive. The ML evaluation of TGR5 agonists showed that the molecular weight (MW), number of hydrogen bond donors (nHDonors), and number of hydrogen bond acceptors (nHAcceptors) were the significant descriptors between active and inactive compounds (Sasaki et al., 2023). This observation can be further compared with drug-likeness principles such as the Lipinski’s Rule of Five, where smaller MW and ideal hydrogen bonding are efficient for pharmacokinetics and biopharmaceutical availability (Brueckner et al., 2024). Nevertheless, some bioactive natural compounds have higher molecular weight (Feher and Schmidt, 2003; Clardy and Walsh, 2004). Besides meeting the Ro5 criteria, natural products with high molecular masses have penetrated the pharmaceutical markets as approved oral drugs (Shultz, 2018; Price et al., 2024).

Active site residues of the crystal structure of TGR5 in complex with its co-crystallised ligand, INT-777, are Leu74, Tyr89, Phe161, Leu166, Tyr240, Thr 243, Leu244, Ser247, Tyr251, Leu262, Leu263, Leu266, and Ser270 as reported by Yang et al. (2020). The docking results show that all the compounds were positioned within the active site of TGR5. It also showed that hydrogen bonding and hydrophobic interactions are important in TGR5 receptor and agonist binding. Particularly, residues such as Tyr240 and Asp348 were predicted to be critical in stabilising the ligand-receptor complex, supporting previous findings on TGR5 activation (Guo et al., 2016). Nevertheless, the flexibility of that binding site remains a major problem for predicting the binding affinities, and to overcome that problem, dynamic studies are required in order to capture the conformational changes of the receptor upon the ligand binding (Mursal et al., 2024).

Molecular dynamics simulations further validated the stability of these interactions, showing that the identified lead compounds formed stable complexes with TGR5 throughout the simulation period. RMSD and RMSF are critical indicators of structural stability and flexibility for a simulation (Ahmad et al., 2020). According to Sindhu and Srinivasan (2015), smaller RMSD values for backbone atoms suggest that the predicted structural models closely match experimental data, indicating higher model accuracy. In contrast, larger RMSD values point to greater deviations and reduced accuracy. This is important in an effort to document the idea that potential drugs do not relinquish their efficiency when exposed to tangible physiology (Brueckner et al., 2024). Simulation studies show compound stability within the TGR5 binding pocket, especially for CNP0417335 and CNP0224616; however, *in vivo* and/or *in vitro* experimental validation is necessary to determining the pharmacokinetic and toxicity profiles of these compounds in biological systems. Intramolecular hydrogen bonds may have stabilised the bioactive conformation of the ligands, which might have led to stronger association observed between the lead compounds and TGR5. These bonds could have acted by lowering the translational and conformational entropy during binding (Davoren et al., 2016), resulting in lower binding energies. Water bridges are also an excellent way to manage protein-ligand complexes; these bridges exist where one or more water molecules are present between the protein and the ligand. Water bridges could have facilitated the formation of a water tunnel in TGR5 during the simulation, as mentioned by Olaposi et al. (2019), leading to the stability of the complexes.

The development of effective TGR5 agonists has been hampered due to gastrointestinal side effects (Zhuo et al., 2024). For instance, INT-777 was found to activate TGR5; however, when tested in the first phases, it was discovered that it poses negative effects on the gastrointestinal tract (Guo et al., 2016). For this reason, there is a need to find selective agonists that do not possess such undesired activity.

Machine learning has enhanced drug discovery and development by increasing efficiency and prediction accuracy. Incorporating big chemical data together with artificial neural network algorithms has enhanced the speed and accuracy of the predictions compared to conventional methods (van Heerden et al., 2023). However, as pointed out in this analysis, existing ML models are vastly dependent on the quality and size of data used in their development, and this has reduced the generality of models in practice. Molecular docking using TGR5 as a subject can also be enhanced by the help of special structural techniques such as cryo-electron microscopy or X-ray crystallography to gain more information regarding the active conformation of the receptor. The integration of these experimental methodologies with MD simulations may improve the reliability of the binding energy predictions and would be beneficial for the design of more selective TGR5 agonists (Brueckner et al., 2024).

## 5 Conclusion

According to this study, new TGR5 agonists for T2D could be developed via ML, MD and MDS techniques. Interestingly, the computational methods studied here illuminate new directions in the search for TGR5 agonists; the actual effectiveness of these approaches remains contingent on the experimental testing of predicted compounds. The integration of these techniques will provide a framework for designing novel TGR5 agonists, and improve the accuracy of identification of lead compounds for T2D treatment.

## Data Availability

The dataset analyzed during the current study as a case study is publicly available at https://github.com/omicscodeathon/tgr5t2d/tree/main/data. The data supporting the results reported in this manuscript is included within the article and its additional files. The generated progress reports are in HTML format and can be viewed using any preferred browser such as Chrome, Safari, Internet Explorer and Firefox. The Project repository which also includes the entire code and other requirements can be downloaded from https://github.com/omicscodeathon/tgr5t2d. The guidelines for implementing this tool and related updates, are available at: https://github.com/omicscodeathon/tgr5t2d/blob/main/README.md.
